# Recovering local structure information from high-pressure total scattering experiments

**DOI:** 10.1107/S1600576721009420

**Published:** 2021-11-23

**Authors:** Anna Herlihy, Harry S. Geddes, Gabriele C. Sosso, Craig L. Bull, Christopher J. Ridley, Andrew L. Goodwin, Mark S. Senn, Nicholas P. Funnell

**Affiliations:** a Department of Chemistry, University of Warwick, Gibbet Hill, Coventry CV4 7AL, United Kingdom; b ISIS Neutron and Muon Facility, Rutherford Appleton Laboratory, Didcot OX11 0QX, United Kingdom; c Department of Chemistry, Inorganic Chemistry Laboratory, University of Oxford, South Parks Road, Oxford OX1 3QR, United Kingdom

**Keywords:** total scattering, high pressure, neutron diffraction, pair distribution function

## Abstract

High pressure is a powerful thermodynamic tool for exploring the structure and the phase behaviour of the crystalline state, and is now widely used in conventional crystallographic measurements. High-pressure local structure measurements using neutron diffraction have, thus far, been limited by the presence of a strongly scattering, perdeuterated, pressure-transmitting medium (PTM), the signal from which contaminates the resulting pair distribution functions (PDFs). Here, a method is reported for subtracting the pairwise correlations of the commonly used 4:1 methanol:ethanol PTM from neutron PDFs obtained under hydro­static compression. The method applies a molecular-dynamics-informed empirical correction and a non-negative matrix factorization algorithm to recover the PDF of the pure sample. Proof of principle is demonstrated, producing corrected high-pressure PDFs of simple crystalline materials, Ni and MgO, and benchmarking these against simulated data from the average structure. Finally, the first local structure determination of α-quartz under hydro­static pressure is presented, extracting compression behaviour of the real-space structure.

## Abstract 2

A method for subtracting the pairwise correlations of a pressure-transmitting medium from neutron pair distribution functions obtained under hydro­static compression is presented and applied to Ni, MgO and α-quartz.

## 1. Introduction

Pair distribution function (PDF) analysis of crystalline materials offers a complementary view to the time-averaged structural information provided by more conventional diffraction experiments. In many instances, material properties can only be understood fully by considering local distortions that cannot be adequately described by an average structure representation. PDF analysis has proved crucial in fully characterizing numerous functional materials, including oxide ion conductors (Scavini *et al.*, 2012 ▸), negative thermal expansion compounds (Chapman *et al.*, 2005 ▸) and the archetypal ferroelectric BaTiO_3_ (Senn *et al.*, 2016 ▸).

There has never been greater provision of facilities capable of making PDF measurements: instruments such as XPDF (I15-1) at Diamond Light Source, UK, 11-ID-B at the Advanced Photon Source, USA, NOMAD at the Spallation Neutron Source, USA, and GEM and POLARIS at the ISIS Neutron and Muon Facility, UK (Connolley *et al.*, 2020 ▸; Ruett *et al.*, 2020 ▸; Neuefeind *et al.*, 2012 ▸; Hannon, 2005 ▸; Smith *et al.*, 2019 ▸) enable high-quality data collection, while also providing average structure measurements. The ability to measure both Bragg and diffuse scattering simultaneously means that both local and average information is encoded within the same scattering pattern, where reciprocal- and real-space information is straightforwardly related by Fourier transform. Thus, in principle, *in situ* experiments can be conducted for local structure measurements using the same methods as for routine powder diffraction. For the most part, this is indeed the case – variable-temperature measurements are carried out in capillary mode with little change to the experimental setup, and *in situ* experiments (for cell cycling, gas flow *etc*.) are designed such that non-sample scattering is reduced as much as possible. The only caveat is that, for generated PDFs to be physically meaningful, parasitic scattering arising from the sample environments must be accounted for by subtracting the scattering signature of the empty equipment (Saha *et al.*, 2015 ▸; Sławiński *et al.*, 2019 ▸; Diaz-Lopez *et al.*, 2020 ▸).

Crystalline materials are often probed by temperature or pressure, to explore their fundamental physical properties via observation of structural changes and phase transitions. Pressure, in particular, can be varied to the extent that it can drive very pronounced structural changes, as crystal structures are forced to rearrange themselves to minimize volume or avoid unfavourable interactions (Moggach *et al.*, 2006 ▸; Eikeland *et al.*, 2017 ▸; Wood *et al.*, 2006 ▸). In general, high-pressure techniques are well established (Besson *et al.*, 1992 ▸; Klotz *et al.*, 1995 ▸) and are no longer the domain of specialist groups, but their use with local structure measurement remains underexplored as significant technical challenges exist. Accessing the gigapascal regime requires very small sample volumes, jeopardizing signal-to-noise levels; however, longer counting times and improved detector efficiencies can mitigate this. More problematic is the complication that arises from the use of a pressure-transmitting medium (PTM) to ensure hydro­static compression – it has its own local structure signal that also changes with pressure. Common media include light organic materials such as methanol/ethanol and pentane/iso­pentane mixtures (Klotz *et al.*, 2009 ▸). This is not such an issue for X-ray experiments, where the scattering of the organic PTM is often negligible relative to that of the sample, and successful PDF measurements have been performed mostly using diamond anvil cells (Chapman *et al.*, 2010 ▸; Wang *et al.*, 2010 ▸), as well as the large-volume Paris–Edinburgh (PE) press, albeit over a very small pressure range (Chapman *et al.*, 2007 ▸). For pressure measurements with neutrons, the PE press is more commonly used but, critically, the PTM must be deuterated to avoid incoherent scattering. The strong coherent scattering of deuterium by neutrons means the PTM contribution to the PDF cannot be ignored.

To date, the only neutron total scattering experiments that have been carried out successfully are those that omit a PTM entirely, *i.e.* non-hydro­static compression, by using a PE press. Amorphous/glassy materials account for the bulk of these studies because they are not particularly susceptible to the effects of strain (Salmon *et al.*, 2012 ▸; Zeidler *et al.*, 2014 ▸). Recently, Playford *et al.* (2017 ▸) showed, using the PEARL instrument at ISIS, that usable PDFs can be obtained for some simple crystalline systems. However, even some of these exhibited signs of strain broadening. Lack of hydro­staticity remains a significant obstacle to measuring local structure in crystalline materials at pressure, and in this article we aim to address precisely this limitation.

Here, we report a method to correct PDFs of crystalline materials measured on the PEARL instrument for the presence of the most commonly used PTM: a 4:1 volume mixture of deuterated methanol and ethanol. We apply an empirical correction based on a combination of molecular-dynamics-informed PDFs and a Metropolis matrix factorization approach to separating sample–PTM scattering contributions (Geddes *et al.*, 2019 ▸; Hua *et al.*, 2021 ▸). The success of our approach is demonstrated through proof of principle for simple crystalline materials – Ni, MgO and α-quartz – for which such measurements have not previously been possible.

## 2. Experimental

### 2.1. Neutron powder diffraction

Crystalline samples of Ni, MgO and SiO_2_, obtained commercially and used as received, were measured on the high-pressure instrument PEARL at the ISIS Neutron Facility (Bull *et al.*, 2016 ▸). Powdered samples were loaded into null-scattering Ti–Zr single-toroid gaskets with a 4:1 volume mixture of perdeuterated methanol:ethanol (ME) PTM (Klotz *et al.*, 2009 ▸). A PE press (Besson *et al.*, 1992 ▸), equipped with zirconia-toughened alumina (ZTA) anvils, was used to apply loads of 2, 25 and 50 tonnes to each sample; in each case pressure was determined from the known equation of state for each material (Chen *et al.*, 2000 ▸; Jacobs & Oonk, 2000 ▸; Angel *et al.*, 1997 ▸). Neutron powder diffraction patterns were collected for a minimum of 9 h each. Analogous data collections were performed for a vanadium pellet and for an ME mixture on its own, also at loads of 2, 25 and 50 tonnes. It was difficult to quantify the mass of ME in each sample loading because it evaporates rapidly, meaning the gasket must be sealed by the PE press to prevent this happening. The gasket and sample were first weighed prior to addition of the ME, to determine their respective masses. Then the complete, now sealed, gasket assembly (*i.e.* sample and ME) was weighed post-compression to obtain an estimate of the ME mass.

### 2.2. Data processing

Data were reduced using the *Mantid* software package (Arnold *et al.*, 2014 ▸), correcting for the effects of attenuation by the ZTA anvils and normalized by a vanadium standard to account for flux profile and detector efficiencies. The gasket and anvil assembly was accounted for by subtracting the scattering from an encapsulated vanadium pellet, measured at equivalent loads to each sample. Total scattering patterns [*S*(*Q*)] were produced by applying a scale factor and *y* offset [*S*(*Q*) × scale + offset] such that *S*(*Q*) → 1 at 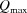 . PDFs were obtained via Fourier transform of the *S*(*Q*) function using the program *StoG*, distributed with the *RMCProfile* package (Tucker *et al.*, 2007 ▸). For each sample, the density and composition were estimated from the difference between the masses of the loaded gasket pre- and post-compression, and were used to normalize PDFs for subsequent treatment. As part of a simplified data treatment, and following established data reduction procedures (Playford *et al.*, 2017 ▸), we did not correct for inelasticity effects, as these were likely to be small for the relatively heavy sample materials, nor did we correct for sample absorption since the attenuation due to the press is likely to dominate over any sample effects. Accordingly, no absorption correction was required to obtain high-quality Rietveld fits (see the supporting information).

PDF modelling and Rietveld refinement were carried out using *TOPAS Academic* (Coelho, 2018 ▸). Simulated PDFs were convolved with a sin(*Q*_max_ *r*)/*r* function where *Q*_max_ = 20.32 Å^−1^, and a d*Q* damping factor of 0.045 Å^−1^ was applied to account for instrumental characteristics.

SiO_2_ PDFs were analysed using ‘large-box’ modelling techniques via the *RMCProfile* software (Tucker *et al.*, 2007 ▸). The refinements used a 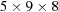 supercell of the Rietveld-refined unit cell, containing 6480 atoms. Eleven independent refinements were carried out for each run to improve the statistical significance of subsequent structural analysis. Potentials-based restraints were applied to the Si—O nearest-neighbour distances and O—Si—O angles to maintain tetrahedral geometry.

### 2.3. Molecular dynamics modelling

Molecular dynamics (MD) simulations were performed using the *GROMACS* package (version 5.1.4, single precision) (Abraham *et al.*, 2015 ▸). The CHARMM36 (Nov18) (Huang *et al.*, 2016 ▸) force field was used to model methanol/ethanol mixtures. The equations of motions were integrated using a leap-frog integrator, with a time step of 2 fs. The van der Waals (non-bonded) interactions were taken into account up to 10 Å, with a switching function bringing them to zero at 12 Å. The particle-mesh-Ewald framework was used to deal with electrostatic interactions (Luty *et al.*, 1994 ▸). To mimic the experimental conditions, we have sampled the isobaric isothermal NPT (constant pressure) ensemble: the stochastic velocity rescaling thermostat of Bussi–Donadio–Parrinello (Bussi *et al.*, 2007 ▸) was used to enforce room-temperature conditions, via a weak coupling constant of 1 ps. The Berendsen barostat (Berendsen *et al.*, 1984 ▸) was employed to apply isotropic pressure on the (cubic) simulation boxes, with a coupling constant of 2 ps. The *P-LINCS* algorithm (Hess, 2008 ▸) was used to constrain O—H bonds. The system contained 1200 methanol molecules and 200 ethanol mol­ecules (9000 atoms in total; molar ratio 6:1, corresponding to a volume ratio of 4.18:1) and was equilibrated at room temperature and 0 GPa for 20 ns. The pressure was subsequently increased in increments of 0.5 GPa to 10 GPa (20 steps in total). At each step, the system was first equilibrated for 10 ns, with the following 10 ns then used to calculate PDFs of the resulting atomistic configuration.

## 3. Results and discussion

### 3.1. Resolving sample and pressure-transmitting medium pair correlations

Once scattering from the PE press is accounted for, the PDF resulting from a variable-pressure hydro­static measurement comprises three components: (i) correlations in the bulk of the pure crystalline material, (ii) correlations within the ME and (iii) crystalline–ME pairwise interactions. We make the initial working assumption that the last of these is in sufficiently low concentration that it can be ignored – we will come to show that the two bulk components adequately describe the whole PDF. We also assume that the structural behaviour of the ME is independent from the sample it is being used to compress. In this way we are able to describe the ME PDF using an analytically derived function, calculable for any pressure between 0 and 10 GPa: the approximate hydro­static range of ME seen experimentally (Klotz *et al.*, 2009 ▸). A non-negative matrix factorization approach is then used to assign relative weights to functions describing the ME and crystalline sample, such that they are straightforwardly separated (Geddes *et al.*, 2019 ▸). We have implemented this procedure in a Fortran90 routine (see the supporting information), which takes variable-pressure, environment-corrected PDFs and user-determined pressure as an input. We outline the procedural steps in more detail below.

### 3.2. Measurements of Ni and MgO

**Figure 1:**
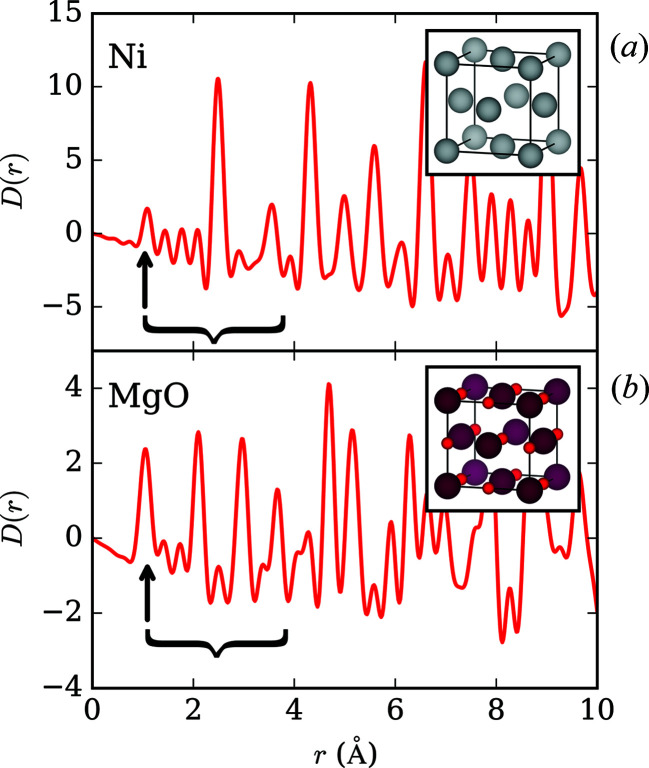
Measured PDFs of Ni (*a*) and MgO (*b*) in the PE press. Black arrows indicate the strongest ME peak and brackets enclose the region over which the more subtle, weakly structured ME contributions extend. Average structure unit cells of Ni and MgO are shown inset.

The average crystal structures of Ni and MgO were confirmed via Rietveld analysis of the measured neutron diffraction patterns – Rietveld fits for all structures at each pressure measured are available in the supporting information. The known equations of state (Chen *et al.*, 2000 ▸; Li *et al.*, 2006 ▸) were used to calculate sample pressures of 0.033 (3), 1.49 (9) and 3.6 (2) GPa for Ni and 0.171 (6), 1.84 (1) and 3.849 (19) GPa for MgO, though we note that the errors on the pressure measurements are probably underestimated. Fig. 1 ▸ shows the lowest-pressure composite Ni and MgO PDFs. We present the PDFs using the *D*(*r*) normalization (Keen, 2001 ▸), because of the clarity it provides for crystalline materials. These PDFs show the composite nature of the sample, with a prominent peak at ∼1 Å arising from C–D and O–D pair correlations in the ME PTM in addition to underlying, unstructured correlations which contribute significantly up to ∼4 Å. Prominent Fourier ripples at low *r* are also present, due to the finite *Q* range of the data. For the purpose of our data processing, described in the next section, it is important to use the *G*′(*r*) normalization instead. Keen (2001 ▸) provides a detailed discussion on these functions.

### 3.3. Variable-pressure modelling of methanol/ethanol PDFs

The analytic function approximating the ME equation of state, relating pressure to the form of the local structure scattering signature, was informed by the MD-simulated atomistic models, where PDFs were generated in 0.5 GPa steps between 0 and 10 GPa. We note that the relevant MD forcefield parameterizations for the methanol and ethanol molecules have been obtained at ambient pressure only. As such, one cannot assume *a priori* that the force field will yield sufficiently accurate results at the high-pressure conditions considered in this work. However, our corrected PDFs, presented later, are indicative of the adequacy of this computational setup.

A notable difference between our simulations and experiment is the substitution of ^1^H with ^2^H in our experiment, in order to avoid the effects of incoherent scattering by neutrons. Whilst force fields for isotopically normal methanol and ethanol are readily available, the same cannot be said for the ^2^H versions of these molecules (Agarwal *et al.*, 2020 ▸). In light of the relatively low resolution of our PDF data, we have accounted for this ^1^H/^2^H difference by shifting the O—H peak position at 0.97 Å (which remains constant across all pressures) by −0.03 Å, corresponding to the difference in H/D covalent bond distances identified by Soper & Benmore (2008 ▸). We did not adjust the C—H bonds, in accordance with observations by Kuchitsu & Bartell (1962 ▸) and Allinger & Flanagan (1983 ▸); in any case the magnitude of the shifts involved is almost negligible, being nearly commensurate with the bin width of our PDFs (0.02 Å).

**Figure 2:**
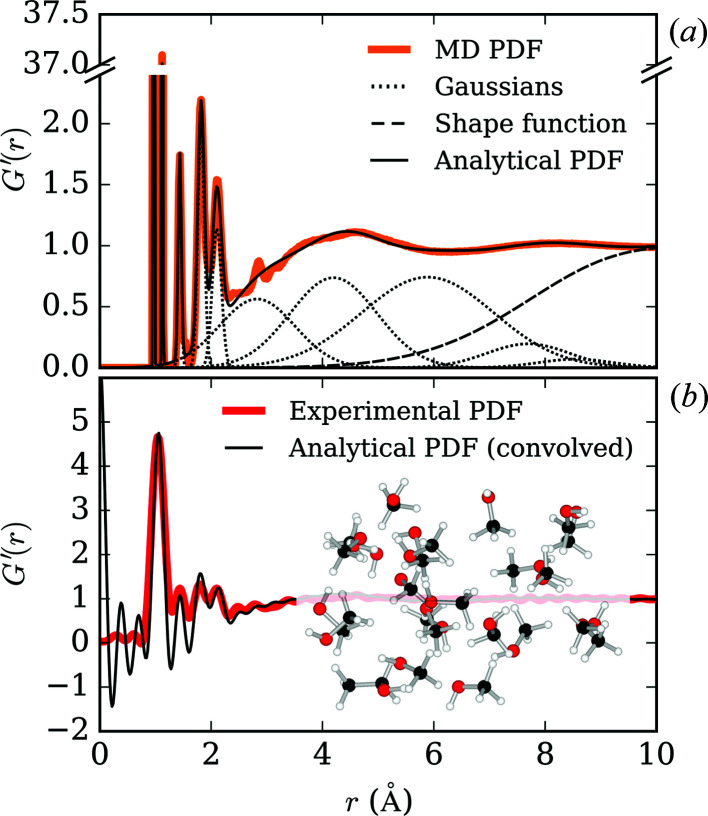
(*a*) Representative MD PDF for ME at 2.0 GPa calculated from MD simulations overlaid with the analytical PDF composed of ten Gaussians and a shape function, described in the main text. (*b*) The same analytical PDF, convolved with sin(*Q*_max_ *r*)/*r* and overlaid with an experimental ME PDF at an estimated pressure of 2.0 GPa.

These MD PDFs [Fig. 2 ▸(*a*)] were used to parameterize the pressure dependence of a series of Gaussian functions that provide a good description of the PDFs at pressures up to 10 GPa. The Gaussians do not have any physical significance; they are simply used as a means to recreate the PDF empirically. Each MD PDF, at pressure *p*, is approximated as the sum of ten Gaussians and an additional function that accounts for the underlying shape of the 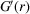 normalization,

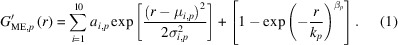




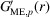
 is the pressure-dependent ME PDF, 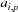 , 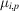 and 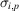 are the pressure-dependent parameters for the *i*th Gaussian, and 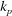 and 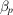 describe the underlying shape of the PDF. We found that when the Gaussian parameters were allowed to vary freely, they displayed a pressure dependence proportional to exp(−*p*). Therefore, we constrained the function to follow this form, in order to reduce the number of parameters required to model the MD PDFs. Each Gaussian and shape function parameter (here we use *x* to denote a parameter 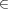 { 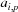 , 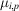 , 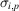 , 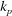 and 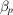 }) then has a pressure dependence

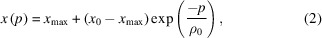

where 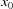 and 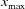 are the parameter values at zero and maximum pressures (10 GPa), respectively, and 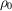 captures the rate of change for these values. Values of 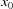 and 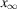 for each parameter, and global 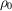 values for 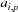 , 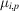 , 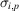 , 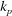 and 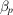 values, were determined by carrying out a simultaneous least-squares refinement against the series of MD PDFs. Thus an end user need only specify a pressure to generate the relevant ME PDF. Plots of all parameters as a function of pressure are available in the supporting information. Finally, the ME PDF is degraded to account for the finite *Q* limit encountered in the diffraction experiment by convolving with a user-defined 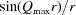 term (*Q*_max_ = 20.32 Å^−1^ for the PEARL experiment, in the examples presented here). The resulting function then more closely matches what is actually measured in the diffraction experiment (see the supporting information). The purely empirical nature of our function to describe the ME means that, in principle, it could straightforwardly be adapted to other PTMs or MD simulations using alternative force fields.

As an assessment of how closely our analytical PDFs represented the local structure of ME, we collected total scattering data of a pure ME sample at applied loads of 2, 25 and 50 tonnes. The PDF measured at a load of 25 tonnes and the corresponding PDF modelled at an estimated pressure of 2.0 GPa are shown in Fig. 2 ▸(*b*).

Though our analytic function does not reproduce subtle features of the MD model in the 2–4 Å region, convolution with sin(*Q*_max_ *r*)/*r* blurs this fine detail. The relatively restricted instrument *Q*_max_ means we need not consider these features; however, this might prove problematic for any neutron instruments with a significantly larger *Q*_max_ value where they could be more clearly resolved. The convolved analytical PDF reproduces all the main features observed in the measured data – sharper peaks at low *r* and broader, less structured correlations at high *r*. The intensity mismatches at low *r* arise from the Fourier filtering that we have applied to the experimental data, using the *StoG* program, specifying a minimum distance below which there are no physical correlations expected (set at 0.8 Å). At high *r*, any differences in intensity are likely to be within error. The difficulty in processing diffraction data from the very weakly scattering ME sample means we can only make a qualitative comparison between our model and experiment, but the reproduction of all the main features of the PDF shows that our model is reasonably good. Further comparisons of the analytic function with the MD-derived and experimental PDFs are available in the supporting information.

### 3.4. Extracting the sample PDF via non-negative matrix factorization

PDFs of composite systems such as amorphous solid dispersions and battery materials have been successfully separated into their constituent components using non-negative matrix factorization (NMF) methods (Geddes *et al.*, 2019 ▸; Hua *et al.*, 2021 ▸). Ordinarily, this approach recovers the relative scattering contributions of individual components to a series of composite PDFs, with continually evolving relative concentrations. The key difference with the problem we face here is that the form of the individual PDFs changes with pressure. We use a modified version of the NMF approach outlined by Geddes *et al.* (and described below) to extract scattering from the sample. To this end we have measured very simple materials (Ni and MgO) with relatively large bulk moduli [*K*_0_ = 177 and 180 GPa, respectively (Zhang *et al.*, 2007 ▸; Kushwah & Shanker, 1998 ▸)], where we anticipate there being little deviation between the local and average structures, and therefore the extracted PDFs can be verified by comparing with ‘small-box’ models generated by the average crystallographic structures.

Once the pressure-dependent ME PDF has been defined, the next step of our procedure is to determine the relative weighting of ME and sample component PDFs for the experimentally measured PDFs. We minimize 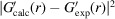 where

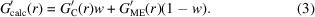




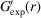
 is the experimentally observed PDF, 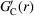 the unknown crystalline sample PDF and *w* the weight of the crystalline component. The sum of the weights is constrained to unity so that the 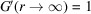 limiting value is maintained and 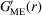 is fixed as the ME PDF calculated via the method outlined above. We also apply a non-negative constraint to both the 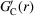 and *w* parameters. Minimization of 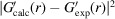 is achieved by a Metropolis Monte Carlo procedure, randomly selecting 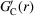 and *w* values at each iteration of the refinement. Simulated annealing is used (Kirkpatrick *et al.*, 1983 ▸), where the acceptance criteria become increasingly strict until convergence occurs and a best fit to the data is realized. When performing the fit, the intensity assigned to the unknown crystalline component is completely unconstrained and so, without guidance, the optimal fit will always result in the unknown crystalline material accounting for the entire PDF. The strongest signature of the ME PDF is found at ∼1 Å, corresponding to C–D and O–D pair correlations. Neither our Ni nor our MgO test case has correlations in this region, so the 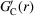 components were only fitted above the shortest atom–atom distance *r*_min_ expected for the sample, and 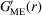 was fitted over the full data range. This highlights a couple of limitations: (i) this approach is unlikely to work well for any sample with a significant number of covalently bound deuterium (or hydrogen) atoms, and (ii) knowledge of the immediate bonding environment in the sample is needed in advance, though this is a reasonable assumption for most experiments covering the 0–10 GPa range.

By design, the refinement preserves a constant sample:ME ratio across a pressure series, though we did examine the ability to recover this trend directly. When the weights are allowed to refine independently of other pressure points, they do show reasonable consistency across the pressure series: 0.55, 0.55 and 0.51 for the Ni sample; and 0.29, 0.30 and 0.29 for MgO. These deviations, though small, are at odds with our experiment – namely, the sample concentration cannot change over the course of compression. The benefit of using the NMF approach to fit to all pressure points simultaneously lies in ensuring that a single, optimal, sample concentration is determined.

Once a best fit has been achieved, and 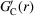 and *w* have been refined, we can correct the as-measured PDF for the PTM contribution. We subtract the calculated ME PDF, weighted by the refined 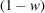 value, from the as-measured PDF, and then correct by multiplying by 1/*w*. All the steps above are performed by a Fortran90 routine which is made available as a supporting information file.

### 3.5. Correction and validation of Ni and MgO PDFs

**Figure 3:**
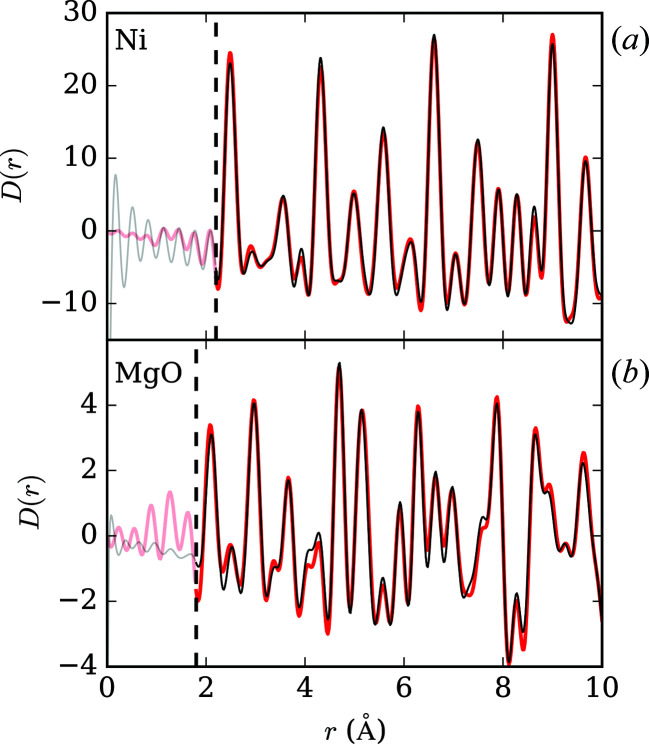
Corrected PDFs (red) for the lowest-pressure PDFs of Ni (*a*) and MgO (*b*), compared with small-box simulated model PDFs (black) derived from average structure starting models. The faded low-*r* regions in each plot indicate where sample peaks are not expected.

We have no means of straightforwardly assessing the corrected PDFs – there are no hydro­static measurements to compare directly against and therefore we have chosen to measure materials where we can anticipate their local structures (free of any interference from ME). Corrected and simulated PDFs should be similar if the effects of ME have been properly removed, and therefore we benchmark the performance of our method against the simulated PDF from the average structure. Fig. 3 ▸ shows the corrected lowest-pressure Ni and MgO PDFs (all measured and corrected PDFs are presented in the supporting information), plotted against fits generated using ‘small-box’ modelling and average crystallographic structures for Ni and MgO ( 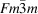 ). The low-*r* regions for both samples are particularly noisy. This is because the sample contribution to the PDF has not been fitted below *r*_min_ and therefore any naturally occurring Fourier ripples have been exaggerated in the corrected PDF and, additionally, the ME PDF imposes large Fourier ripples due to convolution by the sin(*Q*_max_ *r*)/*r* function. Since our method requires that there should be no sample peak overlap with the ∼1 Å ME peak, we accept that any features in the region 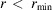 are unrelated to the sample and can be ignored. In both cases, the level of agreement between the simulated and corrected-experiment PDFs is excellent. This shows immediately that, for these simple test cases, the following assumptions hold true: first, that the measured PDF can be treated as two independent components (there are no significant sample–ME correlations present and these can be safely ignored); and, second, that the empirical relationship between the form of the ME PDF and pressure is appropriate.

### 3.6. Local structure of α-quartz under pressure

**Figure 4:**
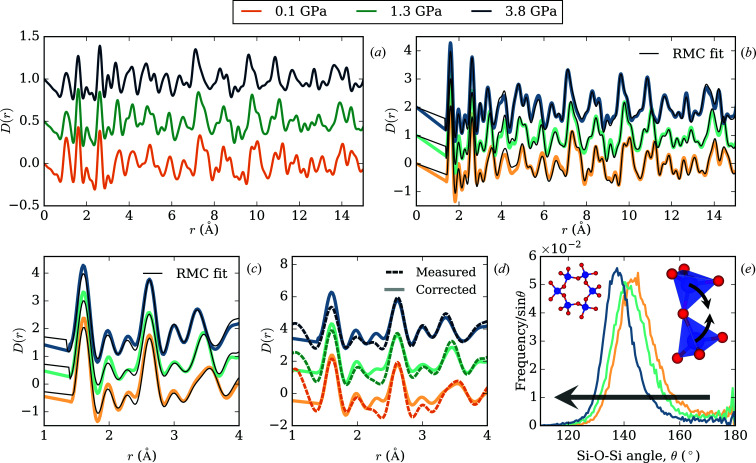
(*a*) As-measured PDFs of α-quartz, offset with increasing pressure. (*b*) Corrected PDFs and their corresponding RMC fits (black lines). Fourier ripples are present and modelled between the first two sample peaks at 1.60 and 2.62 Å. (*c*) Expanded region of (*b*) showing the 1–4 Å region more clearly. (*d*) Comparisons of the as-measured and corrected 1.60 and 2.62 Å PDF peaks, highlighting the effect of ME on the relative peak intensities. The as-measured PDFs have been scaled to aid visual comparison. (*e*) Si—O—Si bond angle distributions from RMC models, corresponding to deformation of the α-quartz structure, with the horizontal arrow indicating angle distribution progression with increasing pressure. The left-hand inset shows the crystal structure connectivity of the SiO_4_ units, and the right-hand inset shows an approximate mode of deformation.

Having confirmed the validity of our approach using simple crystalline materials, the next step is to test a more flexible system and one for which local structure is perhaps not as well described by the average structure. α-Quartz is such a system, with a much smaller bulk modulus (*K*_0_ = 37 GPa) (Liu, 1993 ▸) than Ni and MgO. It has been widely studied using variable-temperature total scattering owing to the fact that the conventional crystallographic analysis presents a geometry and Si—O bond distance that do not accurately describe the true silicate structure (Dove *et al.*, 2002 ▸; Tucker *et al.*, 2000 ▸). Instead, local structure methods have been used in conjunction with ‘large-box’ reverse Monte Carlo (RMC) models to reveal the structural changes and phase transitions driven by temperature changes (Dove *et al.*, 2002 ▸). Local structure measurements under pressure have until now been inaccessible, and therefore we chose to apply the correction described above to α-quartz. The sample was measured on PEARL using the same procedure as for Ni and MgO. Rietveld analysis of the diffraction patterns confirmed the 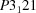 crystal structure (Prakapenka *et al.*, 2004 ▸) at all three pressures (see the supporting information for plots). The absence of pronounced Bragg peak broadening (*i.e*. strain) in the diffraction patterns confirms hydro­static compression of the sample, in contrast to measurements of α-quartz without a PTM (Playford *et al.*, 2017 ▸). This is further supported by the strong structural correlations observed in the PDFs shown in Fig. 4 ▸(*a*), at high *r*, whereas these are damped for samples experiencing strain. The sample pressures at the three applied loads were found to be 0.0766 (11), 1.337 (2) and 3.757 (4) GPa using the refined lattice parameters and equation of state (Angel *et al.*, 1997 ▸). The first PDF peak at ∼1.6 Å, corresponding to the Si—O distance, is sufficiently distinct from the 1 Å ME peak. Corrected PDFs of α-quartz were generated using the NMF approach described above and are shown in Fig. 4 ▸(*b*). The change in the relative intensities of the strong sample peaks at ∼1.60 and 2.62 Å upon subtracting the PTM scattering, shown in Fig. 4 ▸(*d*), illustrates the effect of the more subtle, underlying ME correlations.

RMC modelling, using the *RMCProfile* program (Tucker *et al.*, 2007 ▸), yielded satisfactory fits to the corrected local structure patterns. Though some intensity mismatch is evident, particularly in the 0–1.5 Å region, this is a consequence of refining an overall scaling parameter which helps mitigate against the difficulties in performing an exact normalization of the data at each pressure point. The rigidity of the individual SiO_4_ units is well known and these have been restrained accordingly; the accurate reproduction of all *r*-dependent features in our fit is a strong indication that our data are fit for purpose. Interrogation of inter-tetrahedral angle distributions [>46 900 angles, Fig. 4 ▸(*e*)], extracted from our RMC-refined configurations, shows a contraction of the Si—O—Si angle that appears consistent with the angle compressibility seen in the average structure: 0.012 and 0.011 GPa^−1^, respectively. To the best of our knowledge, these represent the first experimental measurements of the α-quartz local structure under hydro­static pressure. Quartz is one of the most well studied materials by the solid-state community but, until now, analysis of its local structure under pressure has been restricted to computational studies or experimental measurements that are accompanied by strain-induced broadening. Though our RMC models suggest a minimal difference between the local and average angle compressibilities in this instance, the viability of exploring local structure in other hydro­statically compressed, flexible, crystalline systems is exciting.

### 3.7. Method application: opportunities and challenges

We have demonstrated successful recovery of the sample PDFs for the relatively simple systems reported here. Though further tests against more complex systems will be necessary, this is nevertheless an encouraging step towards measuring local structure under hydro­static pressure, using neutrons, especially considering the complexity of deconvoluting the sample and ME scattering signatures. It is important to outline the scenarios where we envisage this method being particularly useful, but also those where it may prove too rudimentary.

The key changes imposed on crystal structures by applying pressure are generally modifications of interatomic distances, as volume is reduced, and possibly phase transitions to new crystal forms. In either case, it is the peak positions of the PDF that are particularly important here, and how they shift as a function of pressure. We anticipate this being the key structural feature a user of this method would be interested in measuring. In most conceivable experiments, the average structure would be known beforehand – this is almost certainly the case for high-pressure experiments, where preliminary measurements to determine the sample’s equation of state would be necessary.

We have already described some situations where our method is less likely to work well. Materials with significant quantities of hydrogen/deuterium atoms will prove problematic because the PDF peak centred around 1 Å is critical for guiding the sample:PTM ratio. Additionally, the difficulty in ascertaining the exact mass of PTM, and thus its density, potentially leads to improper overall scaling of the PDF. This is not such an issue for modelling the PDF, where most modern software packages allow a scale factor to be refined; however it makes determination of coordination numbers unreliable. Again, this is unlikely to be a serious problem for crystalline materials, where the immediate bonding environment will be known from the average structure. Our approach is clearly too limited when it comes to materials possessing only short-range order (*e.g.* glassy/amorphous/liquid materials), where it is challenging to determine their structure; the measurement of coordination numbers is highly important in this field. A comprehensive review of this area is available (Salmon & Zeidler, 2015 ▸).

## 4. Conclusions

Local structure analysis has previously proven crucial in properly identifying structural features/distortions that underpin material behaviour in a wide variety of systems, be it determining local structure mechanisms of battery materials (Malavasi, 2011 ▸; Diaz-Lopez *et al.*, 2020 ▸), defects in metal–organic frameworks (Cliffe *et al.*, 2014 ▸; Coudert, 2015 ▸) or the nature of phase transitions in multiferroic materials (Gilioli & Ehm, 2014 ▸). Thus far, analogous experiments have not been possible for hydro­static high-pressure neutron experiments, restricting exploration of local structure in crystalline materials to near-ambient pressure. The approach we have described mitigates against the PTM limitation and we envisage that this might now make high-pressure PDF measurement of a multitude of crystalline systems viable. An obvious extension of this work would be to explore transferability of the analytic ME function to more complex, flexible materials, *e.g.* frameworks, where the sample PDF would change more rapidly as a function of pressure than seen for the relatively simple systems here. We identify the negative thermal expansion material ScF_3_ (Greve *et al.*, 2010 ▸; Bird *et al.*, 2020 ▸) as a suitable candidate, where the application of pressure could provide insight into its complex expansion behaviour. Our focus on the 4:1 deuterated methanol:ethanol mixture here reflects its common usage, but this method could in principle be applied to other PTMs if their pressure dependence can be straightforwardly expressed as an empirical function.

## Supplementary Material

Non-negative matrix factorization code used to subtract methanol/ethanol scattering from high-pressure neutron PDFs. DOI: 10.1107/S1600576721009420/kc5131sup1.txt

Supporting information. DOI: 10.1107/S1600576721009420/kc5131sup2.pdf
