# Supplementary material for: Recovering local structure information from high-pressure total scattering experiments
Source: J Appl Crystallogr. 2021 Nov 23;54(Pt 6):1546–54. doi: 10.1107/S1600576721009420 (PMC8662973; doi:10.1107/S1600576721009420)
Supplement: Supplementary file 2 [file j-54-01546-sup2.pdf]

JOURNAL OF
APPLIED
CRYSTALLOGRAPHY

Volume 54 (2021)

Supporting information for article:

Recovering local structure information from high-pressure total scattering experiments

Anna Herlihy, Harry S. Geddes, Gabriele C. Sosso, Craig L. Bull, Christopher J. Ridley, Andrew L. Goodwin, Mark S. Senn and Nicholas P. Funnell

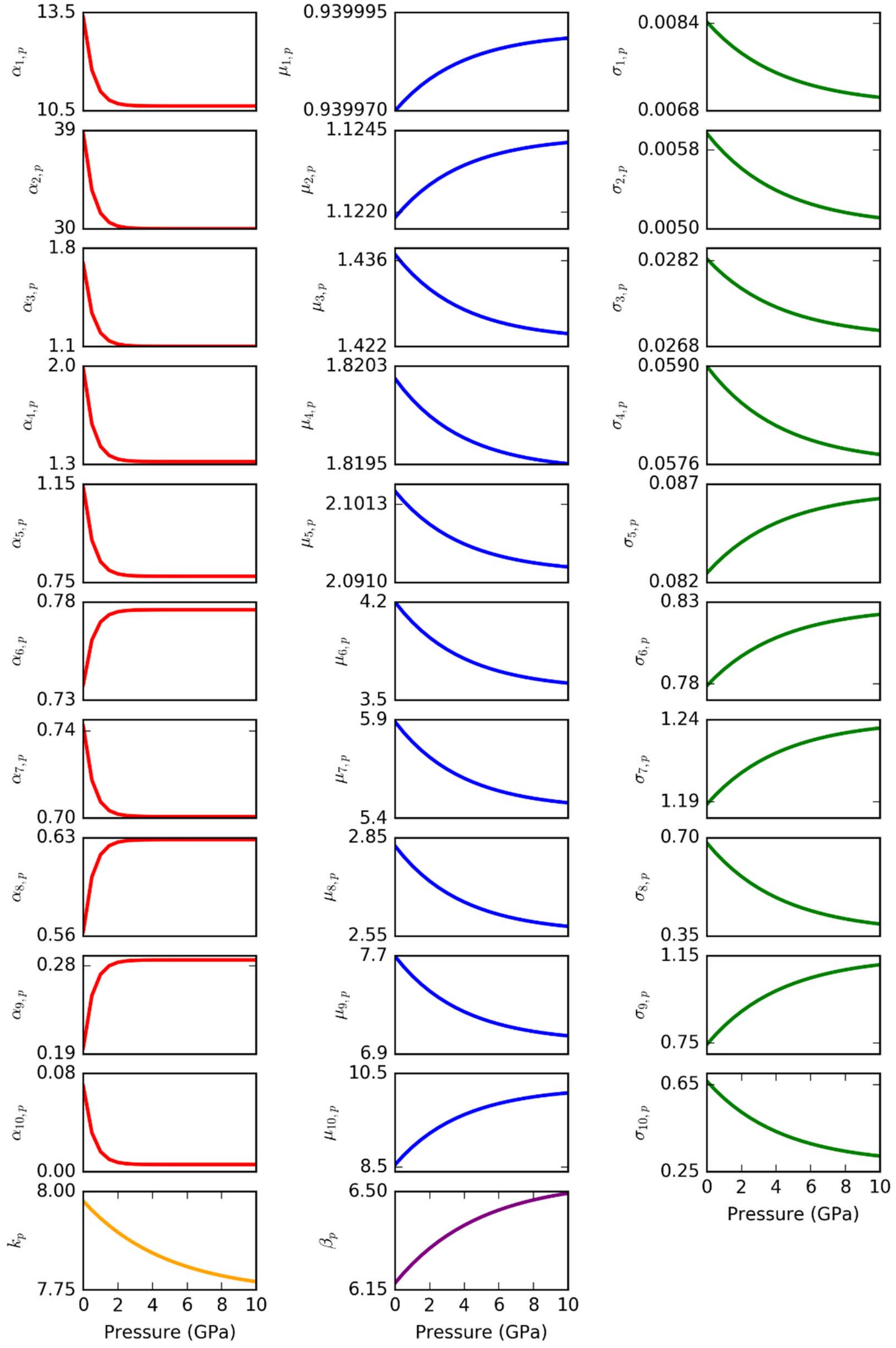

$\alpha_{1,p}$
13.5
10.5
$\mu_{1,p}$
0.939995
0.939970
$\sigma_{1,p}$
0.0084
0.0068
$\alpha_{2,p}$
39
30
$\mu_{2,p}$
1.1245
1.1220
$\sigma_{2,p}$
0.0058
0.0050
$\alpha_{3,p}$
1.8
1.1
$\mu_{3,p}$
1.436
1.422
$\sigma_{3,p}$
0.0282
0.0268
$\alpha_{4,p}$
2.0
1.3
$\mu_{4,p}$
1.8203
1.8195
$\sigma_{4,p}$
0.0590
0.0576
$\alpha_{5,p}$
1.15
0.75
$\mu_{5,p}$
2.1013
2.0910
$\sigma_{5,p}$
0.087
0.082
$\alpha_{6,p}$
0.78
0.73
$\mu_{6,p}$
4.2
3.5
$\sigma_{6,p}$
0.83
0.78
$\alpha_{7,p}$
0.74
0.70
$\mu_{7,p}$
5.9
5.4
$\sigma_{7,p}$
1.24
1.19
$\alpha_{8,p}$
0.63
0.56
$\mu_{8,p}$
2.85
2.55
$\sigma_{8,p}$
0.70
0.35
$\alpha_{9,p}$
0.28
0.19
$\mu_{9,p}$
7.7
6.9
$\sigma_{9,p}$
1.15
0.75
$\alpha_{10,p}$
0.08
0.00
$\mu_{10,p}$
10.5
8.5
$\sigma_{10,p}$
0.65
0.25
0 2 4 6 8 10
Pressure (GPa)
k_p
8.00
7.75
0 2 4 6 8 10
Pressure (GPa)
β_p
6.50
6.15
0 2 4 6 8 10
Pressure (GPa)

Figure S1 Gaussian and shape function parameters ($a_{i,p}$, $\mu_{i,p}$, $\sigma_{i,p}$, k_p and β_p) for the *i*th Gaussian at pressure *p,* used to calculate the methanol/ethanol PDFs. Note the difference in *y*-axis scale between the plots.

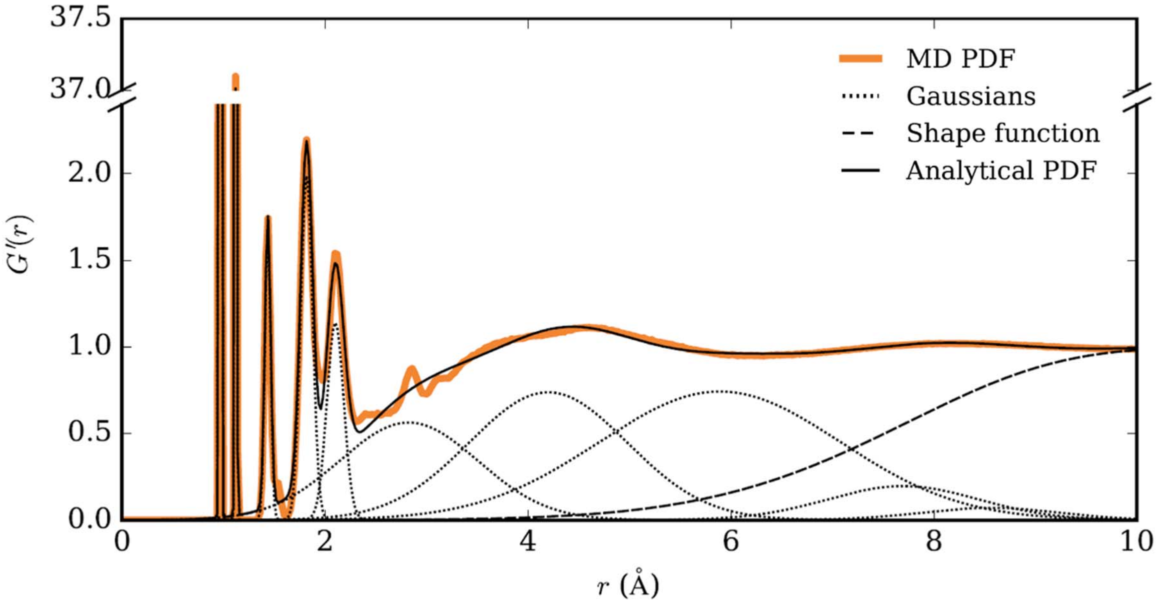

Figure S2 Representative PDF for ME at 2.0 GPa calculated from MD simulations, overlaid with the analytical PDF comprised of 10 Gaussians and a shape function.

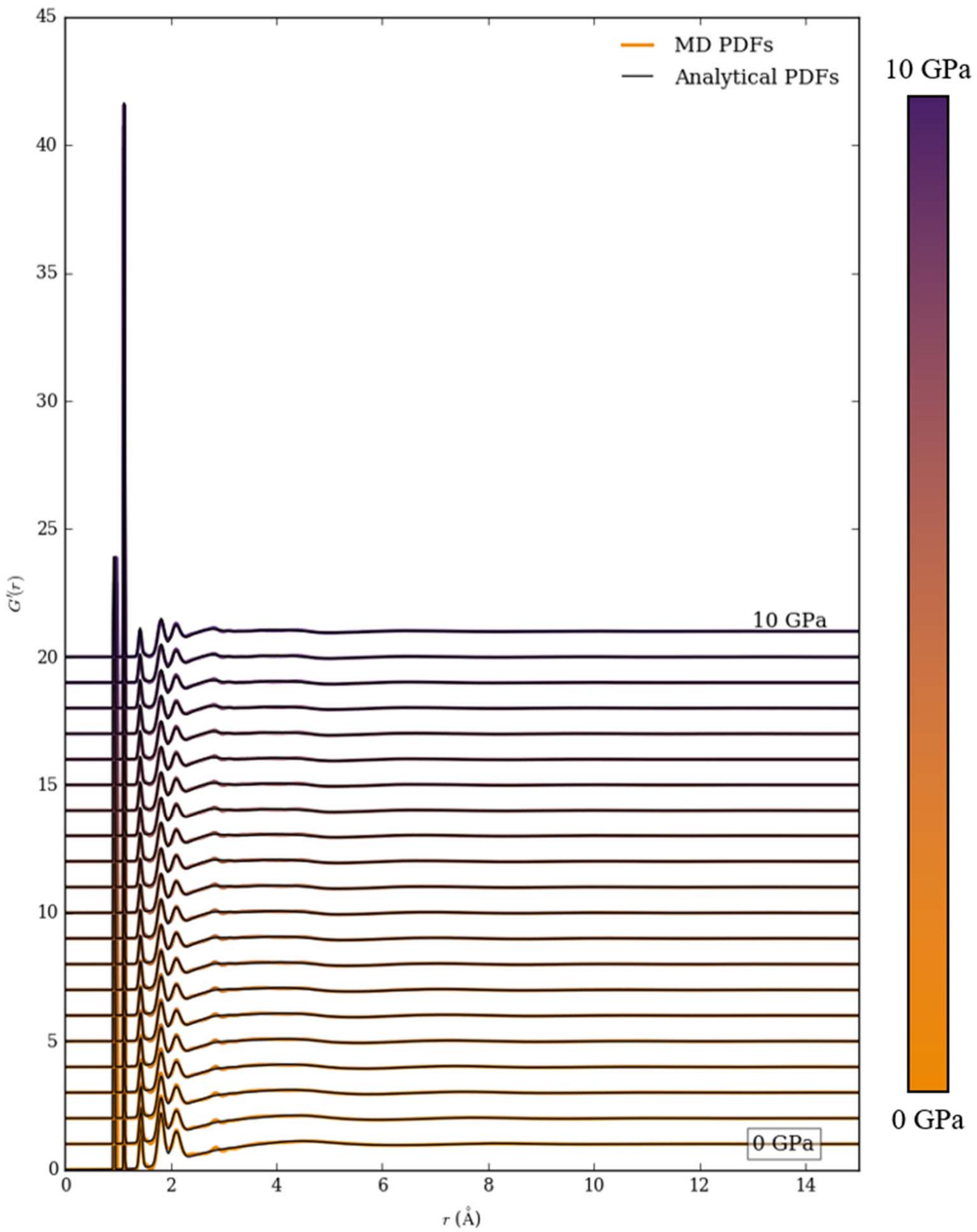

Figure S3 Calculated ME PDFs from each MD simulation and corresponding analytical PDFs, composed of 10 Gaussian peaks and an underlying shape function, at pressures from 0–10 GPa in steps of 0.5 GPa offset in the *y*-direction. The PDFs are free of any instrumental effects—particularly peak broadening which arises due to limited instrumental $Q_{\max}$ values.

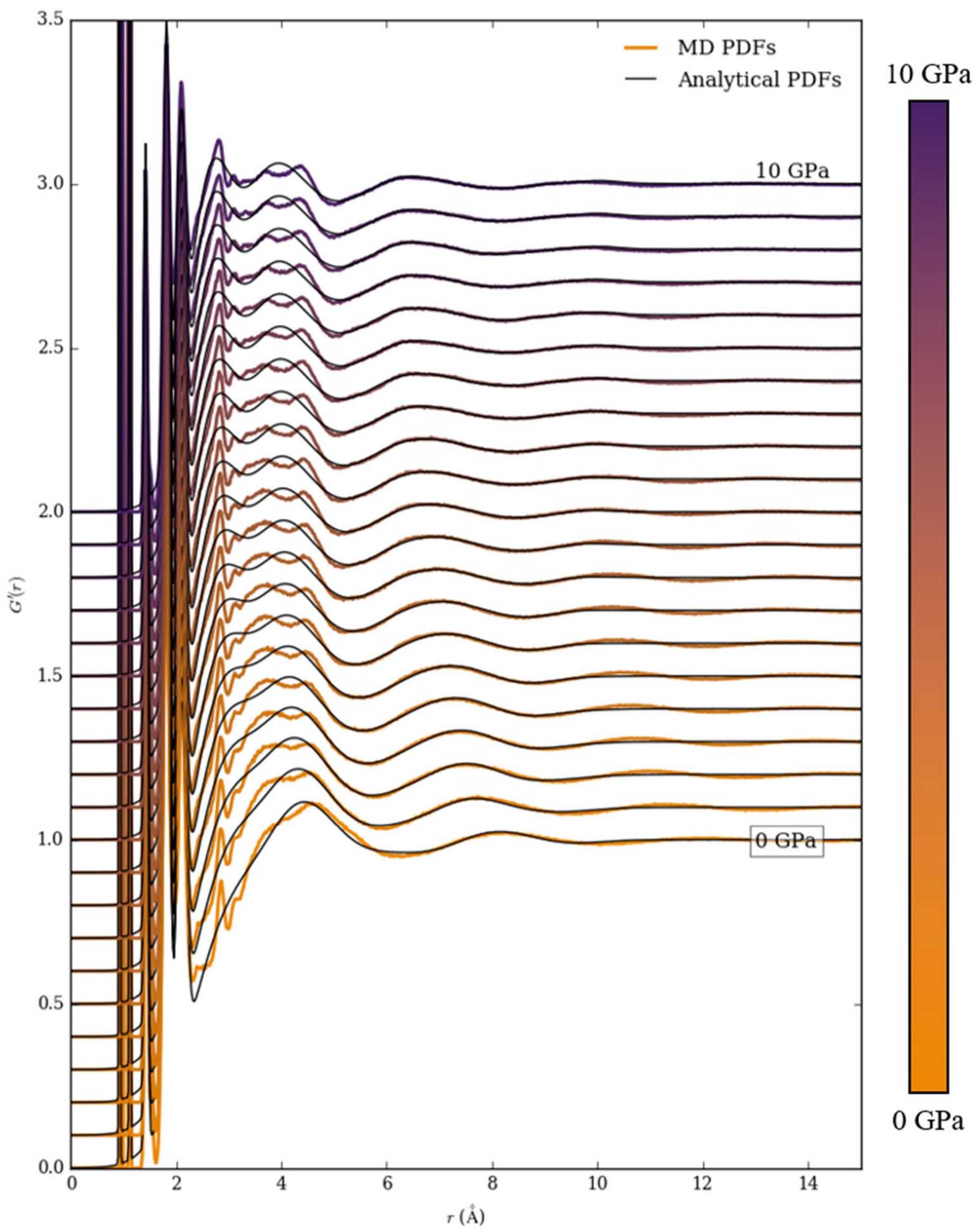

Figure S4 Zoomed comparison of variable pressure MD PDFs (0–10 GPa, as above). Although the 2–6 Å region is not particularly well-described by the modelled analytical PDF, we later show that this detail is lost due to instrumental resolution and that this description is sufficient.

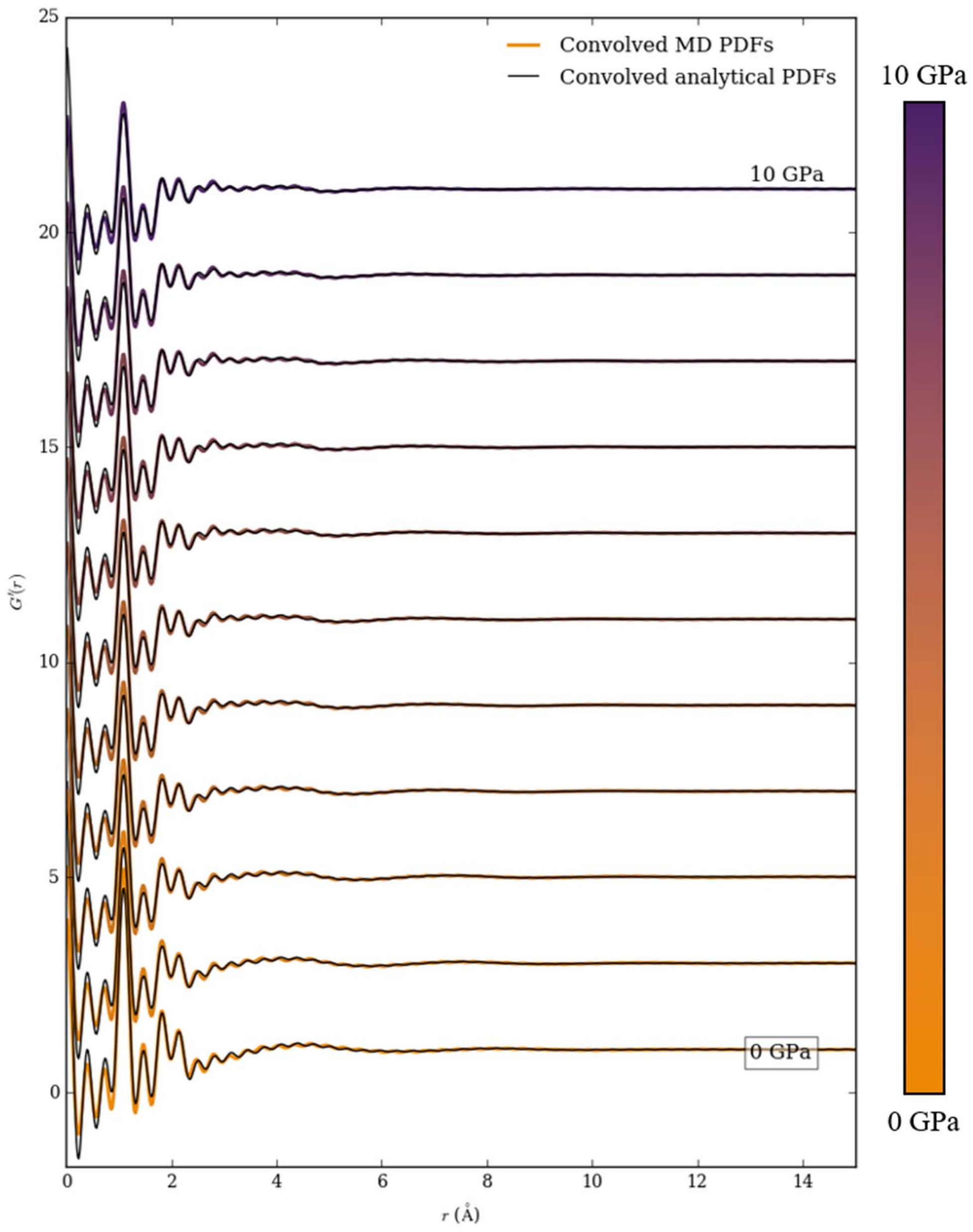

Figure S5 MD PDFs and analytical PDFs, convolved with $\sin(Q_{max}r)/r$, where Q_{max} = 20.32 Å^{-1}. PDFs from 0–10 GPa in steps of 1 GPa are shown, offset in the y-direction. The comparisons of peak positons and intensities show excellent agreement between calculated MD PDFs and analytical PDFs.

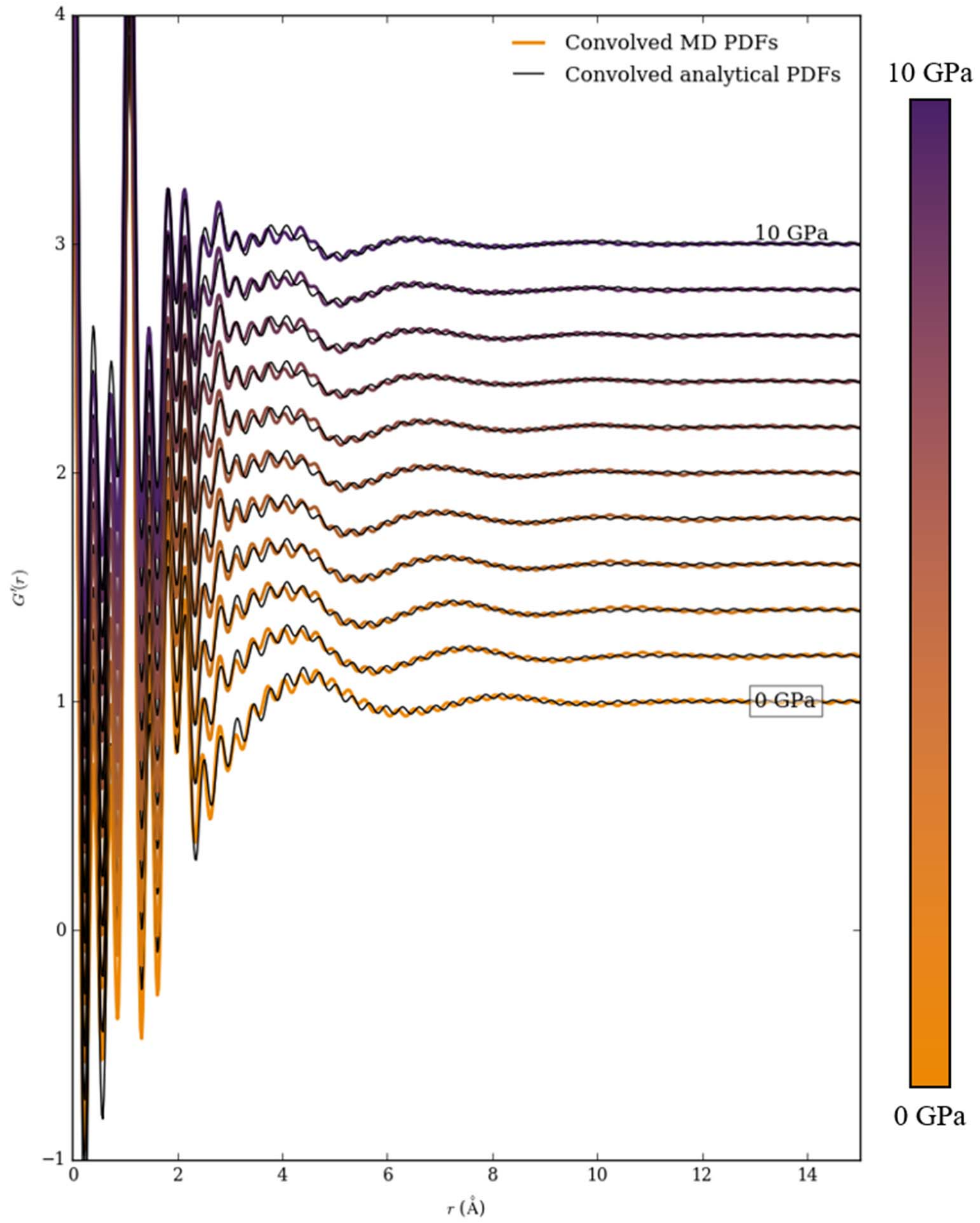

Figure S6 Zoomed comparison of convolved MD and analytical PDFs (0–10 GPa in steps of 1 GPa). Detail in the 2–6 Å region has been lost (comparatively to Figure S2), and the analytical PDFs reproduce these broadened features very well. Ripples in the PDFs are an artefact of convolving to account for a limited Q_{max} value.

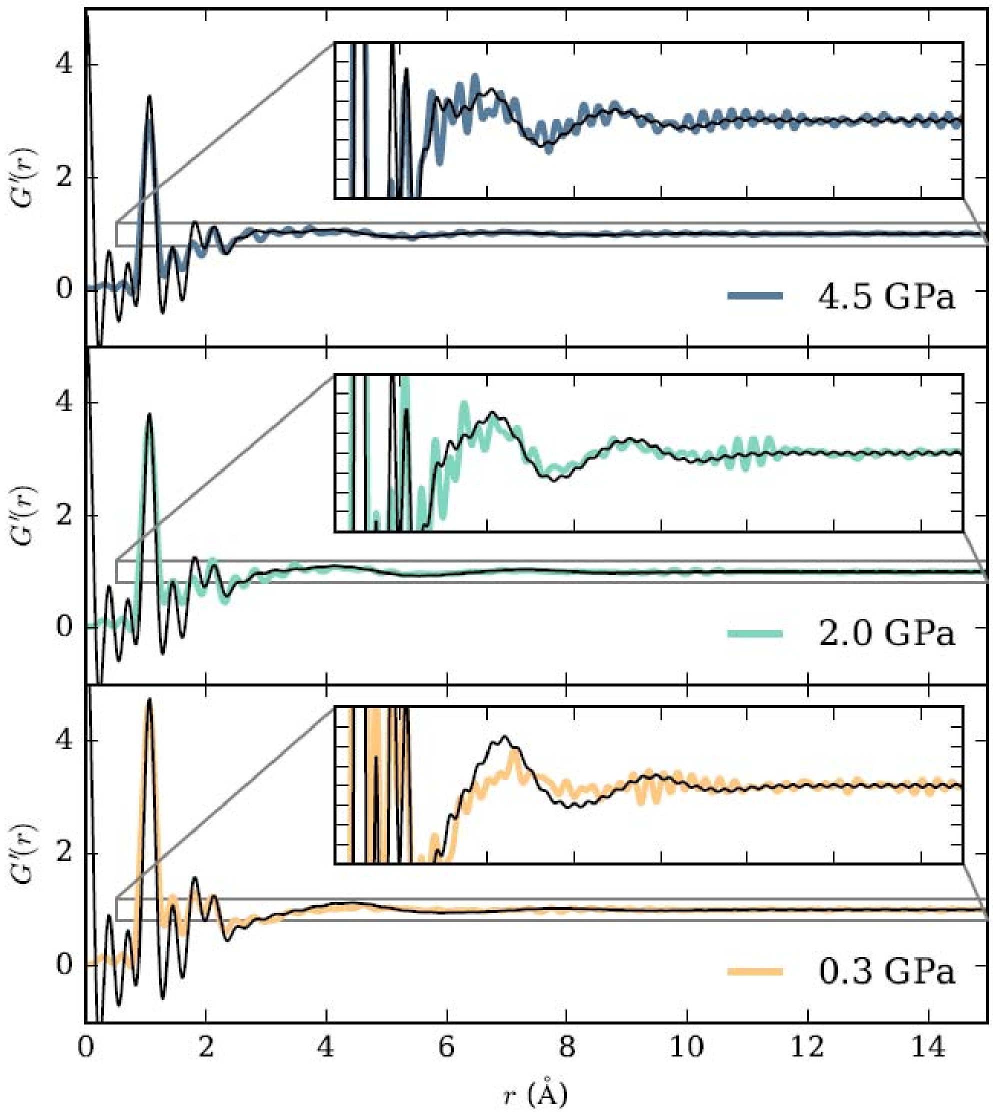

Figure S7 Measured variable-pressure ME PDFs (colours) compared to our analytical ME PDFs (black line). Determining the pressure from an equation of state is not possible with ME and therefore corresponding modelled PDFs were chosen by comparing the 1–6 Å region and selecting the PDF with the best fit. There is very good agreement between the measured and modelled PDFs—peak positions and intensities in the low *r* region are well-replicated, as are broader features at longer length-scales. Small intensity mismatches in low *r* are due to difficulty in data normalisation, and at high *r*, these differences are likely within error of each other.

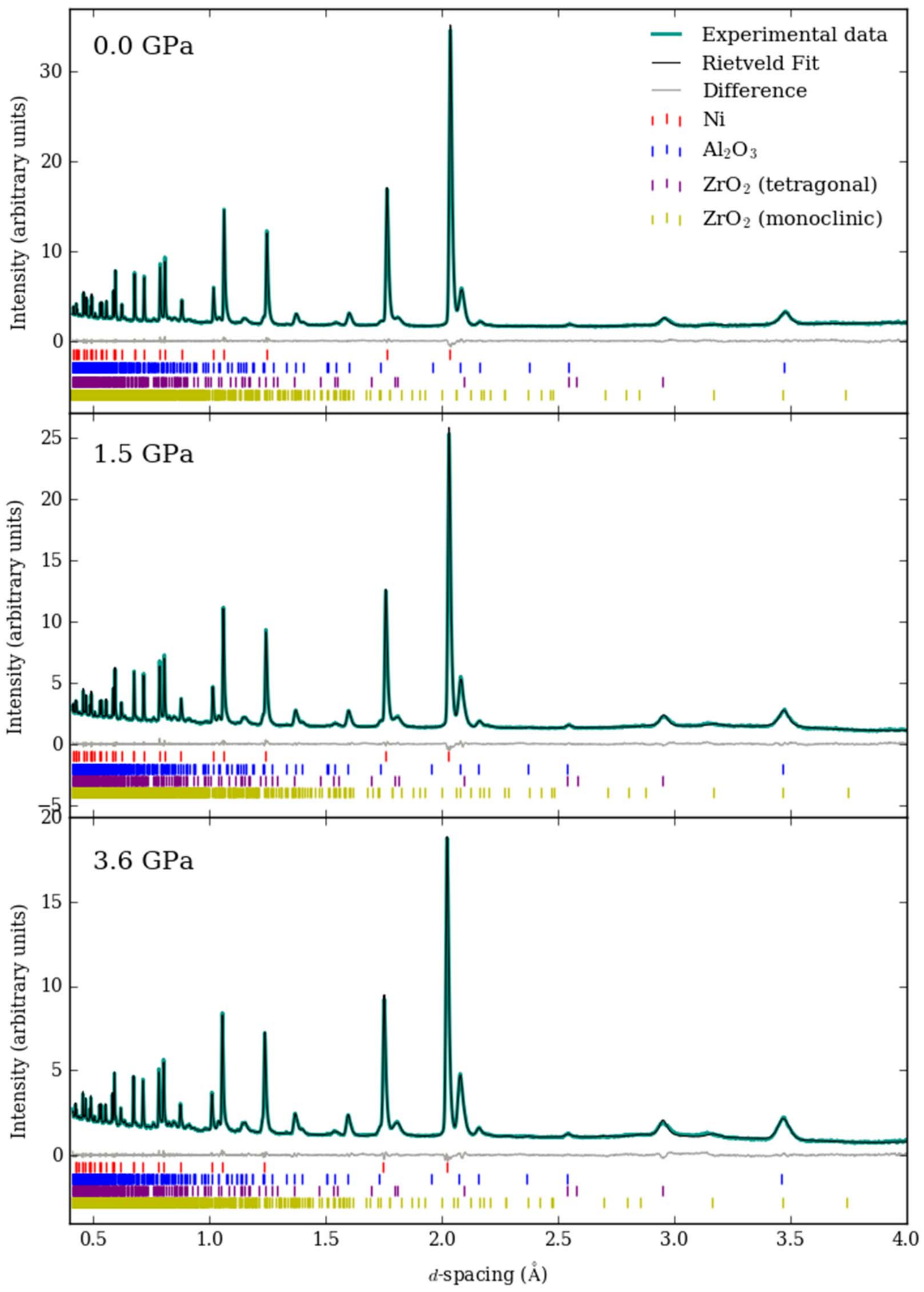

Figure S8 Rietveld fits of diffraction patterns of Ni measured in a Paris-Edinburgh press at pressures of 0.0, 1.5 and 3.6 GPa. Alumina and zirconia peaks are due to scattering from the zirconia-toughened alumina anvils.

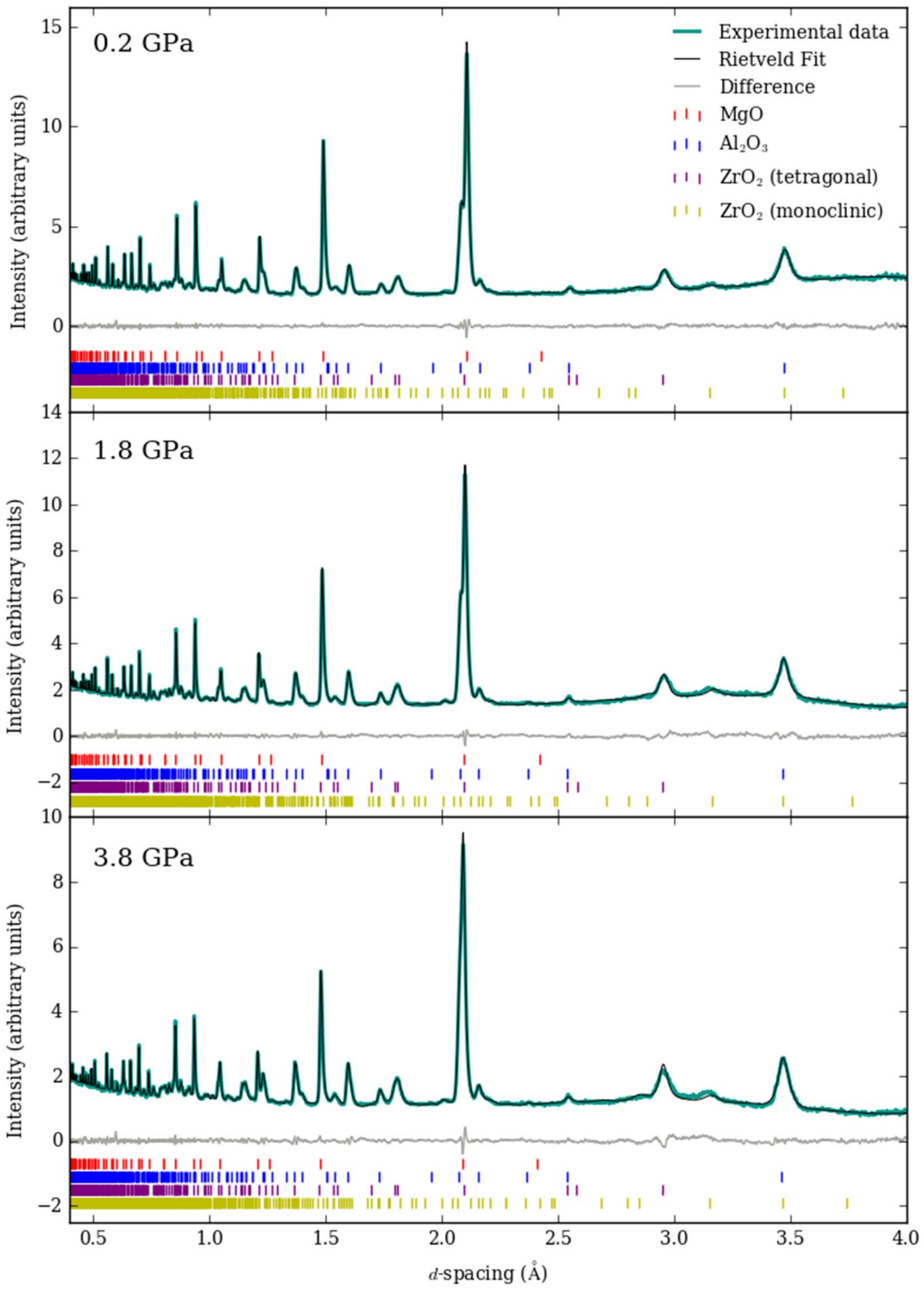

Figure S9 Rietveld fits of diffraction patterns of MgO measured in a Paris-Edinburgh press at pressures of 0.2, 1.8 and 3.8 GPa Alumina and zirconia peaks are due to scattering from the zirconia-toughened alumina anvils.

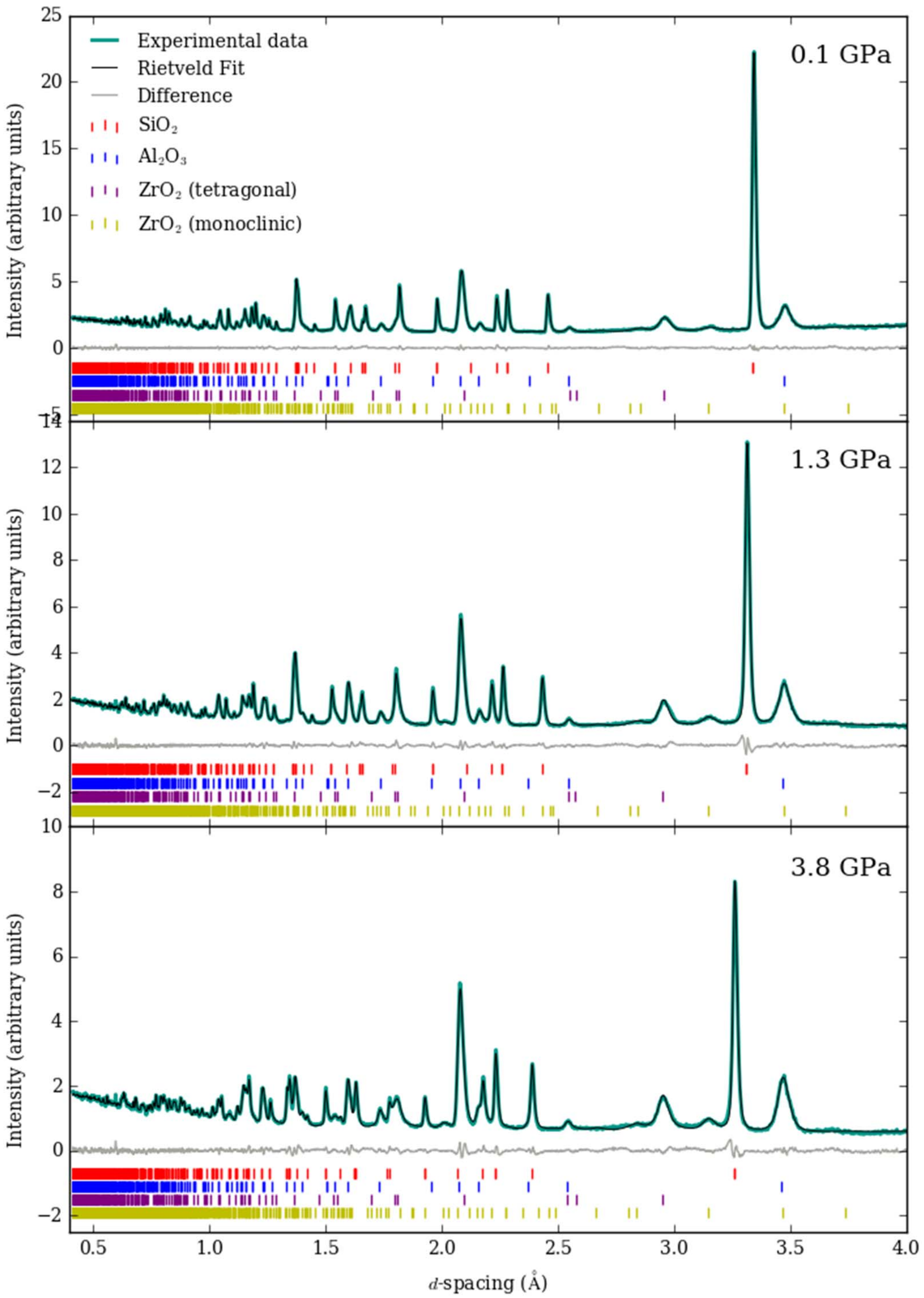

Figure S10 Rietveld fits of diffraction patterns of α-quartz (SiO_2) measured in a Paris-Edinburgh press at pressures of 0.1, 1.3 and 3.8 GPa. Alumina and zirconia peaks are due to scattering from the zirconia-toughened alumina anvils.

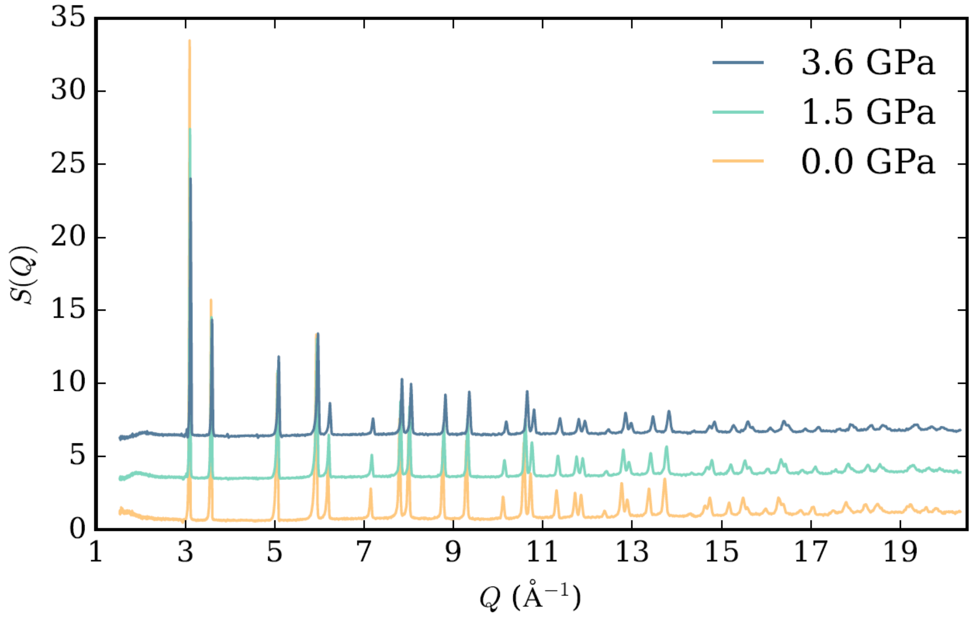

Figure S11 Neutron $S(Q)$ of Ni at 0.0, 1.5 and 3.6 GPa offset in the y-direction.

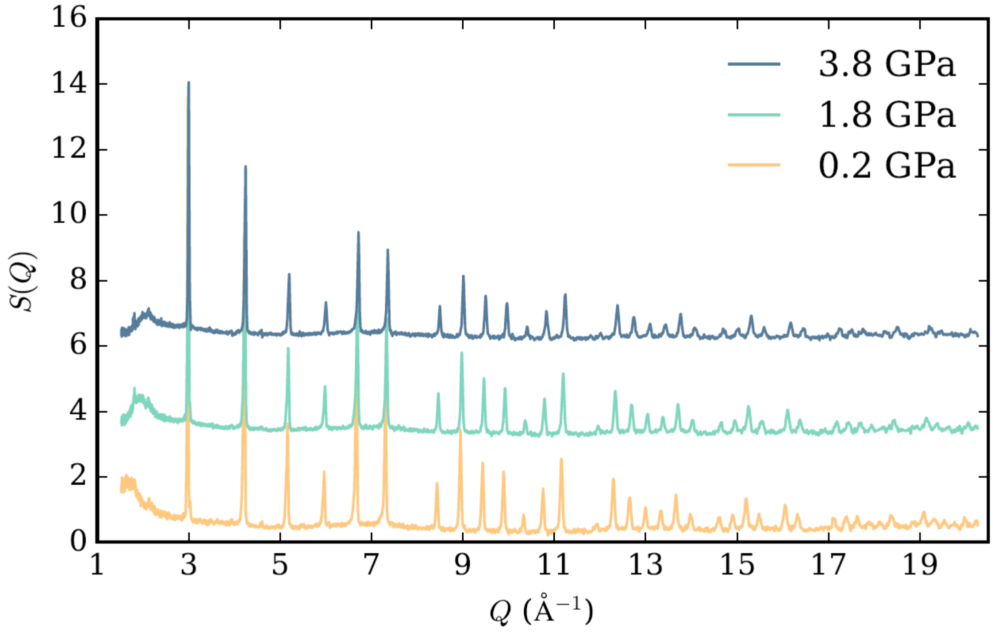

Figure S12 Neutron $S(Q)$ of MgO at 0.2, 1.8 and 3.8 GPa offset in the y-direction.

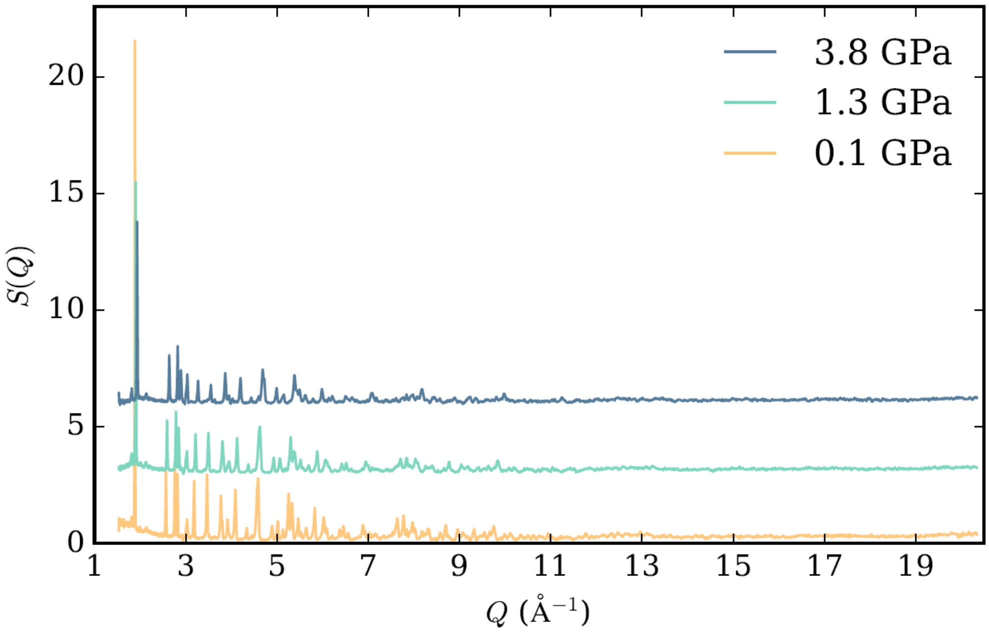

Figure S13 Neutron $S(Q)$ of α-quartz at 0.1, 1.3 and 3.8 GPa offset in the y-direction.

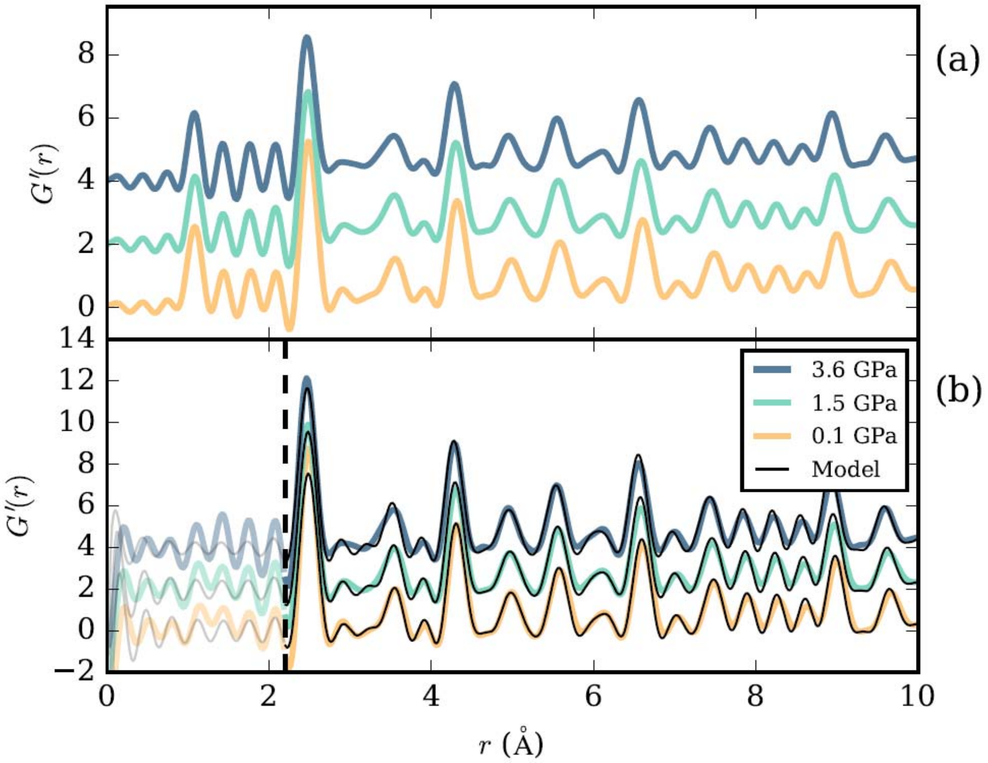

Figure S14 (a) Measured variable-pressure PDFs of Ni. (b) Corrected variable-pressure PDFs of Ni compared to modelled PDFs using refined average structure parameters. The region below 2.2 Å (r_{min}) is omitted during analysis as there are no sample peaks expected here and this region is particularly noisy due to ME PDF-imposed Fourier ripples.

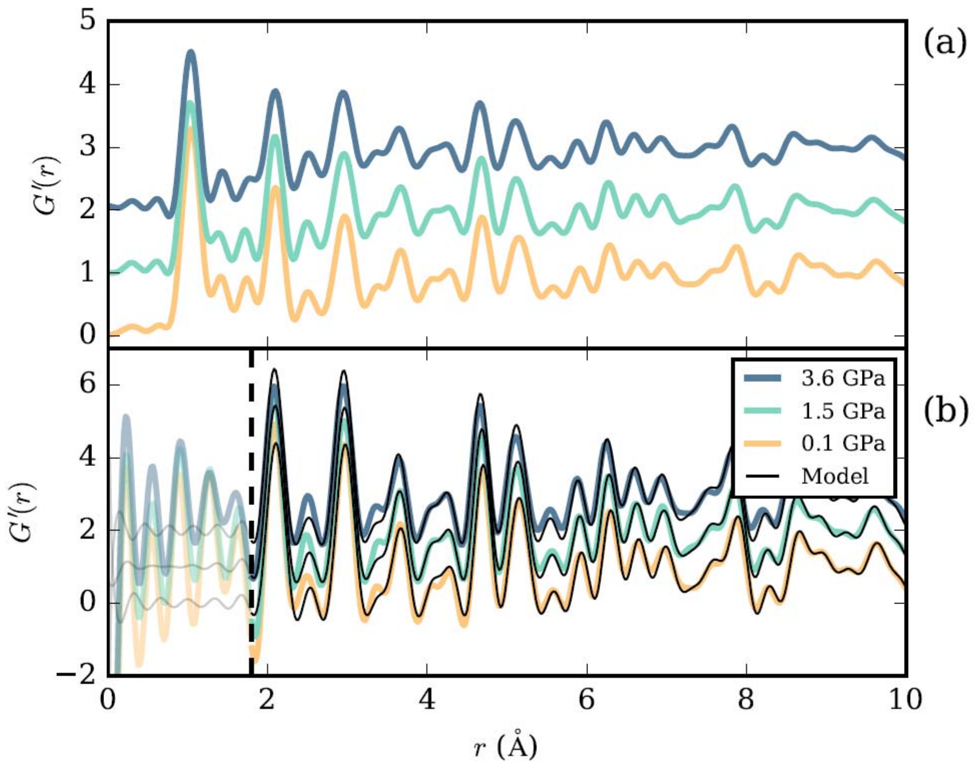

Figure S15 (a) Measured variable-pressure PDFs of MgO. (b) Corrected variable-pressure PDFs of MgO. The region below 1.8 Å (r_{min}) is omitted during analysis as there are no sample peaks expected here and this region is particularly noisy due to ME PDF-imposed Fourier ripples. All features modelled by the average structure are reproduced in our corrected PDFs.

S1. ***RMCProfile*** settings

RMCProfile configurations were generated using 6 × 9 × 8 supercells of the Rietveld-refined average structure unit cells. 11 independent runs were performed for each pressure point, running for 3 days each, ensuring fit convergence. Closest approach (CA) constraints were applied between atom pairs; their respective values were informed by peak tails in the PDFs at each pressure. Additional potentials-based restraints (invariant with pressure) were used to preserve approximate tetrahedral connectivity of the SiO_4 units. The values used are given in Table S1 below. Angle and distance energies were unavailable for SiO_4 units in the *RMCProfile* manual; the values used here were informed by other similar, rigid, tetrahedral groups. The reader is referred to the *RMCProfile* manual for details on the implementation of the energy parameter.

Table S1 Atom–atom constraints used in *RMCProfile*.

Atom pair	CA 0.1 GPa	CA 1.3 GPa	CA 3.8 GPa
Si–Si	2.00	2.00	2.00
Si–O	1.45	1.40	1.35
O–O	2.40	2.35	2.38

**Atoms**	**Distance / Å**	**Angle / °**	**Energy / eV**
Si–O	1.60	N/A	2.00
O–Si–O	N/A	109.5	7.60
